# Comparative Transcriptome Analysis of Hens' Livers in Conventional Cage vs. Cage-Free Egg Production Systems

**DOI:** 10.1155/vmi/3041254

**Published:** 2025-03-21

**Authors:** María Paula Herrera-Sánchez, Roy Rodríguez-Hernández, Iang Schroniltgen Rondón-Barragán

**Affiliations:** ^1^Poultry Research Group, Laboratory of Immunology and Molecular Biology, Faculty of Veterinary Medicine and Zootechnics, Universidad del Tolima, Altos de Santa Helena, Ibagué 730006299, Tolima, Colombia; ^2^Immunobiology and Pathogenesis Research Group, Laboratory of Immunology and Molecular Biology, Faculty of Veterinary Medicine and Zootechnics, Universidad del Tolima, Altos de Santa Helena, Ibagué 730006299, Tolima, Colombia

**Keywords:** hens, production systems, stress, transcriptome

## Abstract

Different conditions of production systems including stocking density, thermal conditions, and behavior restriction can have a significant detrimental effect on the health and performance of laying hens. The conventional cage system is one of the systems that have been reported to cause stress problems in birds, due to social and behavioral stress. Emerging technologies have facilitated a deeper understanding of animal responses to various scenarios and can be an additional tool to conventional ones to assess animal welfare, where transcriptomic analysis has the potential to show the genetic changes that occur in response to stress. According to this, the aim of this work was to characterize the liver transcriptome of hens housed under two egg production systems (conventional cage and cage-free). Liver tissue from Hy-Line Brown hens housed in conventional cage (*n* = 3) and cage-free (*n* = 3) production systems at week 80 of age was processed using the Illumina platform to identify differentially expressed genes with a padj < 0.05. Regarding the differentially expressed genes, 138 genes were found, of which 81 were upregulated and 57 downregulated. Some of the genes of interest were *TENM2, GRIN2C,* and *ACACB*, which would indicate greater fat synthesis in the liver of caged hens. The enriched KEGG pathways were DNA replication and the cell cycle. In conclusion, it was identified that the cage production system may influence DNA replication and the cell cycle since the genes related to these terms were found suppressed, which would indicate cellular instability.

## 1. Introduction

Egg production has provided high-quality animal protein worldwide [1]. To meet the increasing demand, various production systems, such as conventional cages (CCs), enriched cages, aviaries, and grazing systems, have been developed, seeking to balance efficiency and profitability [2, 3]. However, CCs remain the predominant method, although they raise concerns for animal welfare due to the stress they cause in birds [4]. Alternative systems, such as cage-free (CF), are designed to improve welfare conditions by allowing natural behaviors, such as foraging and nesting [4, 5]. Despite the advantages of these systems in animal health and welfare, they also present challenges, such as increased risks of disease due to contact with soil and other vectors [6, 7].

Factors such as stocking density in CC egg production systems aggravate the effects of stress in birds, affecting their behavior, physiology, and productivity [8]. Activation of the hypothalamic–pituitary–adrenal (HPA) axis is key in the stress response, influencing survival and long-term health, with glucocorticoids, particularly corticosterone (CORT), playing a crucial role [9, 10]. Overcrowding causes aggressive behaviors and elevates CORT levels, which reduces immune function and reproductive performance and increases ROS production that causes damage to DNA, lipids, and proteins, affecting bile acid biosynthesis and causing metabolic and immune system alterations [8, 11–15]. Moreover, the liver, crucial for metabolism and energy production, is particularly susceptible to oxidative stress, which impairs its function [16].

Monitoring hen welfare through the physiological stress biomarkers is necessary to ensure their welfare and optimize productivity in poultry production [17]. Several techniques have been used to determine the stress response, among which the ELISA test to quantify CORT levels in serum, plasma, or feathers is often considered the gold standard due to its direct correlation with adrenal activity [18]. The use of CORT as a stress biomarker in chickens has shown inconsistencies, which could be due to cross-reactivity with other glucocorticoids, and it is necessary to explore alternative methods such as gene expression analysis to assess stress responses in laying hens [18, 19]. The use of omics technologies, such as transcriptome analysis, allows the evaluation of a wide range of genes under specific conditions, facilitating the identification of molecular mechanisms and unknown genes or isoforms [16, 20]. These techniques also provide a deeper understanding of cellular physiology and its regulation, allowing the capture of gene expression dynamics and new events such as alternative splicing variants [21]. According to this, this work aimed to characterize the liver transcriptome of hens housed under two egg production systems (CC and CF).

## 2. Materials and Methods

### 2.1. Study Population

The present study was carried out by processing tissue samples obtained from a previous research project in which laying hens from a commercial egg production farm were housed in two different production systems, CC and CF, in the same location, complying with the corresponding sanitary and legal requirements [22]. The commercial production was located in Ibagué, Department of Tolima, between the coordinates 02°52′59″ and 05°19′59″ north latitude, and 74°24′18″ and 76°06′23″ west longitude, at an altitude of 1250 m above sea level, with an average temperature of 25°C, and sampling was carried out during the period 2018-2019.

Under commercial conditions, 60,000 Hy-Line Brown pullets were raised from one day of age. These were housed in cages of 76.22 × 66.05 m, with a density of 16 pullets per cage (315 cm^2^/bird). The pullets were maintained under the same health, management, and feeding conditions until 15 weeks of age. Subsequently, at week 16, the same pullets were transferred to two different housing systems, CC and CF, on the same farm, until 82 weeks of age. A total of 45,000 hens were housed in a CC system, in a pyramidal battery of vertical cages in Californian-type facilities (40 × 40 × 40 cm). Each battery had four floors and nipple drinkers, and the house had a ventilation system with cooling panels. In this house, four hens were located per cage (450 cm^2^/hen). Likewise, 720 hens were evaluated in 15 replicates of 12 cages each (48 birds/replicate), for a total of 180 cages evaluated in the CC system.

The CF production system evaluated was a barn-type system. This system had a deep rice husk bedding floor, mesh-fenced houses, and natural ventilation. Each house had communal nest boxes, which were two-tiered wooden structures, with 10 nests measuring 40 × 40 × 40 cm per tier (5 hens/nest), and one 4 m perch per tier. The 14,850 hens were housed in two houses at a stocking density of 1111 cm^2^ per bird. These houses were divided into 15 enclosures, which were used as replicates for the CF system, with 990 hens per enclosure. Birds were provided with water and feed, and a lighting schedule of 14 h of light and 10 h of darkness was used. Diets were identical for the two production systems (CC and CF) and were formulated following the recommendations of the Hy-Line Brown Layer Management Guidelines. Health and nutritional management were carried out in accordance with the poultry company policies.

### 2.2. Samples

For the transcriptome analysis, liver tissue samples were used from the CC system (*n* = 3) and CF system (*n* = 3) at 80 weeks of age. Approximately 0.5 g of liver was kept in tubes containing RNAlater (Thermo Fisher Scientific, United States), and they were stored at −20°C.

### 2.3. RNA Extraction and Sequencing

The tissue samples were sent for processing at Novogene (Sacramento, United States) for total RNA extraction and sequencing. RNA was isolated from each sample using the TRIzol:chloroform method following the manufacturer's instructions. The integrity and purity of the extracted RNA was verified with the Agilent 2100 Bioanalyzer (Agilent Technologies, United States). Samples with RIN (RNA integrity number) values > 6 were used for library construction. Messenger RNA (mRNA) was purified from tRNA using Oligo (dT)-bound magnetic beads. After fragmentation, random hexamer primers were used to synthesize the first strand of cDNA, and for the nondirectional library, the second strand of cDNA was synthesized using dTTP. The constructed library was used to perform sequencing on the Illumina NovaSeq 6000 platform, with a 150-bp (paired-end) sequencing strategy.

### 2.4. Quality Control, Mapping, Counting, and Batch Effect Correction

Quality control of raw reads was performed using FastQC (version 0.12.1), and removal of adapters and reads with quality < Q20 was performed using Trim Galore (version 0.6.7) with the paired-end option [23, 24]. The filtered reads were then used to map to the chicken reference genome (GRCg7b) using HISAT2 (version 2.2.1) [25]. The mapped reads were used to generate a counting matrix using FeatureCounts from the Subread package (version 2.0.3) [26]. After counting the mapped reads, batch correction was performed on the reads using the RUVg function that uses negative control genes to estimate unwanted variation factors from the RUVSeq package (version 1.38.0) in R (version 4.4.0) [27]. The corrected count files were used for differential gene expression (DEG) analysis.

### 2.5. DEG Analysis

The DESeq2 package (version 1.44.0) in R (version 4.4.0) was used to detect DEGs between CC and CF groups [28]. The *p* value was obtained by the Wald test and was corrected by multiple testings using the Benjamini–Hochberg (BH) method [29]. All genes were considered as DEGs with a padj < 0.05 and a log2 fold change < −1 and > 1. Graphs were generated using the pheatmap (version 1.0.12) and ggplot2 (version 3.5.1) packages in R (version 4.4.0).

### 2.6. Gene Ontology (GO) Enrichment Analysis and Kyoto Encyclopedia of Genes and Genomes (KEGG) Pathway Enrichment

The list of obtained DEGs was used to perform GO and KEGG pathway enrichment analysis with the ClusterProfiler package (version 4.12.0) and the organism database org.Gg.eg.db (version 3.19.1) in R (version 4.4.0) [30]. GO and KEGG analyses were considered significant with a q value (qvalue) < 0.05.

### 2.7. Quantitative Polymerase Chain Reaction (qPCR) Validation

To verify the reliability of the expression profiles of the transcriptome data, some genes from the DEG list were evaluated by qPCR. Six tissue samples of liver tissues (*n* = 6) from hens housed in CC and CF at week 80 of age were used for this validation. Total RNA (tRNA) was extracted from liver samples using RNA-Solv reagent (Omega Bio-Tek, United States), and cDNA was synthesized using the GoScript Reverse Transcriptase System Synthesis Kit (Promega, United States).

In addition, Luna Universal qPCR Master Mix reagent (New England BioLabs, United States) and the QuantStudio 3 Real-Time PCR system (Thermo Fisher Scientific, United States) were used. Thermal profiling included an initial denaturation step at 95°C for 1 min, followed by 40 cycles of denaturation at 95°C for 15 s and annealing at 60°C for 30 s. Subsequently, melting curve analysis was performed at 95°C for 1 s, 60°C for 20 s, and a continuous increase of 0.15°C/second up to 95°C. Samples were processed in duplicate. Relative gene expression was quantified using the 2^−ΔΔCt^ method [31], using the *ACTB* gene as a reference [32]. Primers were designed to evaluate the relative gene expression of the genes angiopoietin-like 4 (*ANGPTL4*), DnaJ heat shock protein (HSP40) family member C12 (*DNAJC12*), acetyl-CoA carboxylase beta (*ACACB*), bradykinin B1 receptor (*BDKRB1*)*, Solute carrierfamily 6 member 12 (SLC6A12), Minichromosome maintenance complex component 2 (MCM2),* and 3*(MCM3)* (Table 1).

### 2.8. Statistical Analysis

Determination of data normality using the Shapiro–Wilk test was performed. To determine the differences in gene expression between the CC group and the CF group, *t*-test or Mann–Whitney test was used, depending on the normal distribution of the data, with results expressed as mean ± SEM. Analyses were performed using GraphPad Prism v 10.3.1, with a *p* value < 0.05 being set to consider differences statistically significant.

### 2.9. Ethical Approval

This study was approved by the Bioethics Committee of the University of Tolima, following Colombia's laws and according to Act 007-2020 [22].

## 3. Results

### 3.1. Quality Analysis, Mapping, and Removal of Batch Effects

RNA samples were obtained from the liver of hens housed in CCs (CC1, CC2, and CC3) and in CF systems (CF1, CF2, and CF3). These samples presented a RIN value between 6.4 and 7.6, considered adequate for library preparation.  Table 2 shows the raw reads obtained, with an average of 50,283,646 reads for the CC group and 50,510,734 for the CF group, with a sequence length of 150 bp. In addition, in the CC group reads, 93% of the bases had a Phred value of 30, as in the CF group reads, indicating a 99.9% accuracy in the assignment of nucleotides at each position. Similarly, the sequence quality with a Phred of 20 in both groups was on average 97% (Table 2). After filtering the sequences, between 49,256,784 and 50,544,382 reads were obtained in the CC group, while between 49,646,028 and 51,271,664 reads were obtained in the CF group.

**Figure 1:**
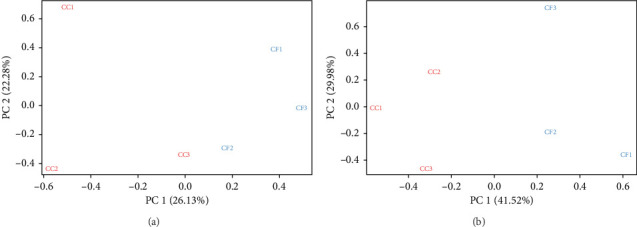
Comparison of sample grouping before and after removing batch effects: (a) before batch effect correction; (b) after batch effect correction. Samples from the livers of hens housed in conventional cages (CC1, CC2, and CC3); samples from the livers of hens housed in cage-free systems (CF1, CF2, and CF3).

The mapping results (%) showed that between 95.75% and 96.59% of the reads were aligned to the reference genome (GRCg7b), where 87.94%–89.05% of the reads mapped uniquely (Table 3). Additionally, the results obtained using featureCounts showed that between 71.3% and 74.3% of the reads were assigned to annotated genomic features of the genome. Furthermore, in the principal component analysis (PCA), a batch effect was observed (Figure 1(a)), where samples CC3, CF1, CF2, and CF3 were grouped on the right side and samples CC1 and CC2 on the left side, evidencing a separation in the PC1 axis (26.13%). After removing the variations, the corrected data (Figure 1(b)) showed that the samples are grouped by group, with a variability in the PC1 axis of 41.52%.

### 3.2. DEG in Liver Tissue From CC Versus CF Hens

**Figure 2:**
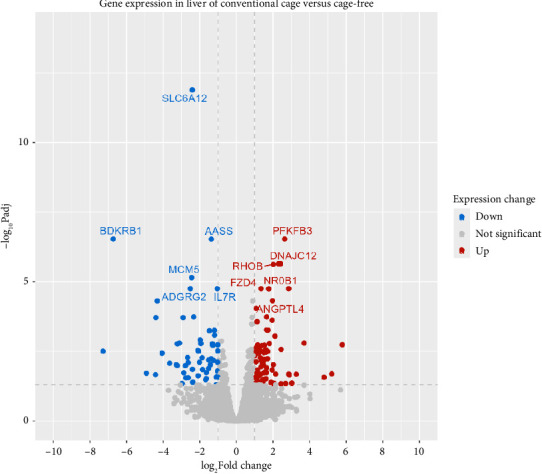
Volcano plot of DEGs in liver tissues from conventional cage (CC) versus cage-free (CF) hens. Vertical dotted lines indicate log2FoldChange < −1 and log2FoldChange > 1, and the horizontal dotted line indicates −Log10padj (0.05).

Using DESeq2, a total of 1641 differentially expressed genes were detected out of 14,574 (*p* value < 0.05), among which 326 were upregulated (log2FoldChange > 1) and 232 were downregulated (log2FoldChange < −1). Of the 1641 genes, 209 genes had padj values < 0.05. 138 DEGs were considered in this study of which 81 had a log2FoldChange > 1 and 57 had a log2FoldChange < −1 (Figure 2). Furthermore, among the 138 genes, 118 were categorized as protein-coding genes, 18 lncRNA genes, and 2 novel genes.

**Figure 3:**
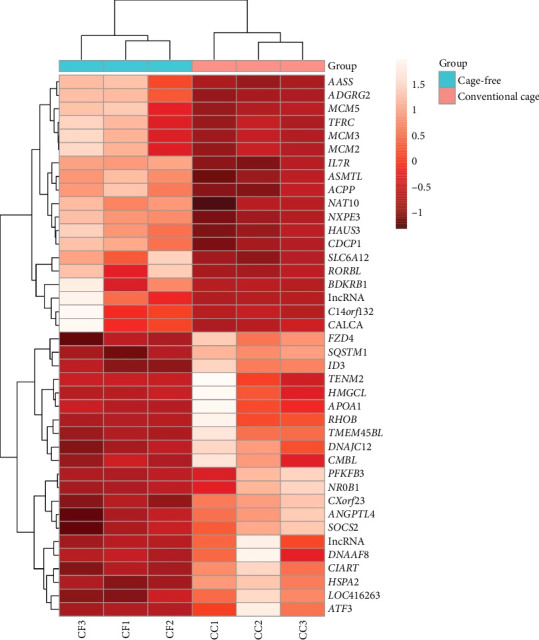
Heatmap of 40 genes differentially expressed in the liver of conventional cage (CC) versus cage-free (CF) hens. The bar indicates the gene expression level according to its mean (*Z*-score).

In Figure 3, the heatmap with hierarchical clustering of 40 genes is shown, evidencing a difference in the gene expression pattern between the CC and CF groups, which is corroborated by the upper dendrogram. In summary, teneurin transmembrane protein 2 (*TENM2*), NMDA-type glutamate ionotropic receptor subunit 2C (*GRIN2C*), *ACACB*, Type 45 transmembrane protein (*TMEM45BL*), and noggin (*NOG*) were the genes with the highest log2FoldChange values, indicating upregulation. Also, other genes of interest that showed positive log2FoldChange values were *ANGPTL4*, 6-phosphofructo-2-kinase/fructose-2,6-bisphosphatase 3 (*PFKFB3*), circadian-associated transcriptional repressor (*CIART*), Ras homolog family member B (*RHOB*), *DNAJC12*, caspase recruitment domain family member 10 (*CARD10*), and suppressor of cytokine signaling 2 (*SOCS2*). Furthermore, some genes that were found to be downregulated were cutaneous T-cell lymphoma-associated antigen 1 (*CTAGE1*), *BDKRB1*, SH3 domain-containing ring finger 2 (*SH3RF2*), *SLC6A12*, and interleukin 7 receptor (*IL7R*) (log2FoldChange < −1). The results of the detected genes (padj < 0.05) are shown in supporting table S1.

### 3.3. GO Enrichment Analysis and KEGG Pathway Enrichment

**Figure 4:**
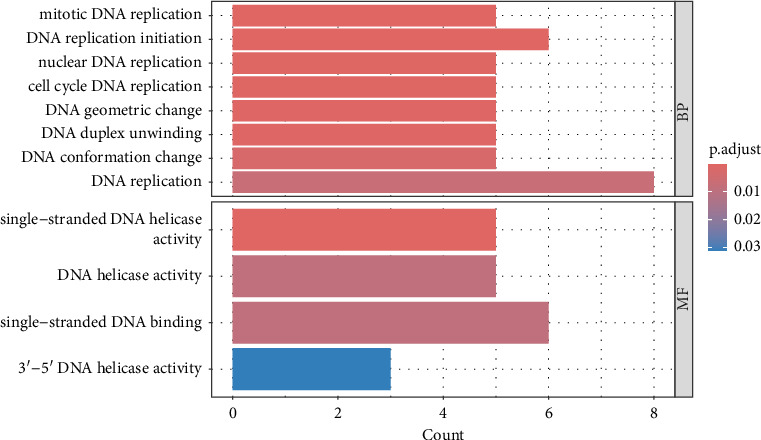
Gene ontology (GO) enrichment. The colors in the figure correspond to the adjusted *p* value (padj), and red indicates significant enrichment. BP: biological processes and MF: molecular function.

**Figure 5:**
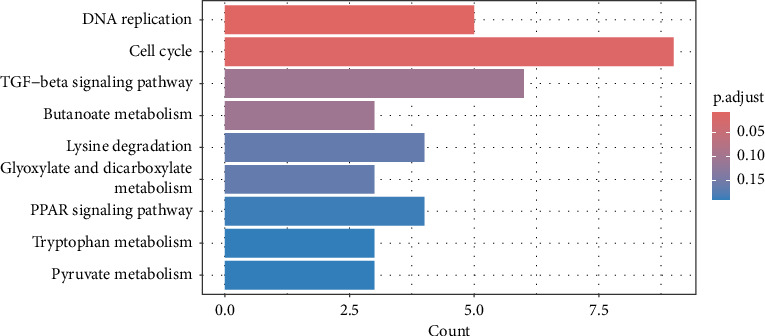
Enriched KEGG pathways of differentially expressed genes (DEG) (padj < 0.05). Colors in the figure correspond to the adjusted *p* value, and red indicates significant enrichment.

**Figure 6:**
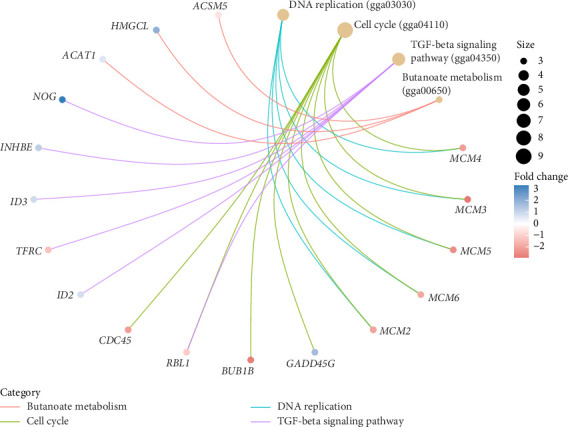
Cnet plot of enriched KEGG pathways (*p* value < 0.05). Size: the number of DEGs belonging to the enriched KEGG pathway; fold change: fold change between liver samples from CC housed hens versus CF hens.

The results revealed a total of 22 GO-enriched terms, distributed as follows: 17 in biological process (BP), one in cellular component (CLC), and four in molecular function (MF). In BP, the enriched terms in the liver of caged versus CF hens were *mitotic DNA replication, DNA replication initiation, nuclear DNA replication, cell cycle DNA replication, DNA geometric change, DNA duplex unwinding, DNA conformation change, DNA replication, rhythmic process, circadian regulation of gene expression, DNA-templated DNA replication, double-strand break repair, double-strand break repair* via *homologous recombination, recombinational repair, autophagy of mitochondrion, circadian rhythm,* and *DNA recombination* (FDR qvalue < 0.05) (Figure 4). These terms are generally related to DNA replication and repair, cellular maintenance, and circadian rhythms. Regarding CLC, the most enriched term was *MCM complex*. In MF, the enriched terms included *single-stranded DNA helicase activity, DNA helicase activity, single-stranded DNA binding,* and *3′-5′ DNA helicase activity* (FDR qvalue < 0.05) (Figure 4). For KEGG pathways, two enriched pathways were identified: *DNA replication* and *cell cycle* (FDR qvalue < 0.05) (Figure 5).  Figure 6 shows the genes related to these two pathways, which are downregulated (*CDC45, RBL1, BUB1B, MCM2, MCM3, MCM4, MCM5, MCM6*), except *GADD45G*. The enrichment results were consistent between GO terms and KEGG pathways (Supporting tables S2 and S3).

### 3.4. Validation of Transcriptome Results

**Figure 7:**
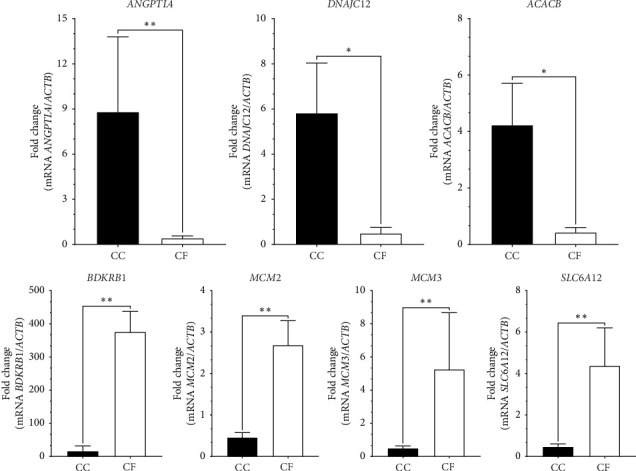
Relative gene expression of the genes used to validate the transcriptome results in liver tissues of laying hens at 80 weeks of age. The beta actin gene (*ACTB*) was used as a reference gene. ⁣^∗^*p* value < 0.05; ⁣^∗∗^*p* value < 0.01. CC: conventional cage; CF: cage-free.

**Figure 8:**
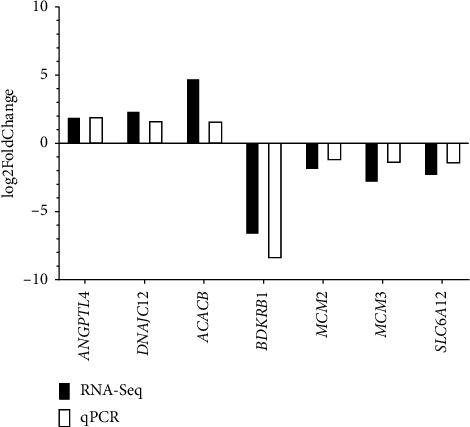
Comparison of log2FoldChange of transcriptome data (RNA-Seq) versus qPCR.

To validate the transcriptome expression profiles, the genes evaluated were *ANGPTL4, DNAJC12, ACACB, BDKRB1, MCM2, MCM3,* and *SLC6A12*. These genes were found to be differentially expressed between the CC and CF groups according to the qPCR results obtained (Figure 7). Moreover, Figure 8 shows similar expression patterns between the results of log2FoldChange from transcriptome data and qPCR data, corroborating that the transcriptome data were reproducible.

## 4. Discussion

CC egg production systems allow the maintenance of large numbers of animals in small spaces [34]. However, animal density in production is a crucial factor, as it affects production as well as animal welfare by causing physiological stress [35]. In birds, the liver plays a fundamental role in lipid metabolism, as it is the site where lipogenesis occurs and lipids are transported to the egg yolk to nutrify the embryo and maintain homeostasis during stress [16, 36]. However, the stress caused by the high density of animal storage generates oxidative stress, where glutathione (GSH) levels increase in response to maintaining the intracellular oxidation–reduction balance; moreover, fat accumulation, inflammation, and organ malfunction are produced [37–39]. In this context, a comparative analysis of the liver transcriptome of hens housed in CC systems versus CF systems was carried out at week 80 of age.

Transcriptome is a widely used technique in various animal species to identify the effect of factors such as temperature, feed, or drugs on the gene expression of animals [40]. In this study, the factor to be evaluated was the CC versus CF egg production system. Initially, an average of 50,283,646 and 50,510,734 reads were obtained for the CC and CF groups, respectively. In addition, after removing adapters and low-quality reads, more than 49,256,784 reads were obtained for both groups (CC and CF). These reads are higher than those obtained in other studies that report between 7 and 10 million reads and 20.7–42 million reads [40–42]. Additionally, the batch effect correction was performed, which was identified by the grouping of the data in the PCA. It has been reported that batch effects may be due to factors external to the experimental design, such as different sample handlings and different batches of reagents, among others [43]. In transcriptome and metabolome analyses of chickens from different samples such as brain, blood, and liver, this correction has been performed, allowing the removal of unwanted variations that could affect subsequent analyses [44–46].

In the present study, 138 differentially expressed genes were identified in the liver of hens housed under the CC production system versus the CF system at week 80 of age. The genes that were upregulated with the highest log2FoldChange values were *TENM2*, *GRIN2C*, *ACACB*, *TMEM45BL*, and *NOG*. In the case of the *TENM2* gene, it has been reported that it participates in adipogenic differentiation, and in the case of the chickens fed with different diets, a higher expression of this gene in the liver has indicated a greater absorption of carbohydrates and fats in the groups with low-protein diets [47]. Furthermore, this gene has been associated with excess food and fluid intake, where its inactivation causes a decrease in the levels of triacylglycerols (triglycerides), which are the most common storage fats and, therefore, the most important source of fatty energy [48, 49]. Under stress conditions, feed consumption in animals is reduced, but fat deposition increases, and under chronic stress, hepatic triglyceride content and plasma levels of very low-density lipoproteins (VLDL) increase, preparing the hepatic system as a method to adapt [16]. According to the results obtained in this study and what was mentioned above, the overexpression of this *TENM2* gene in the liver tissue of the CC group could indicate an increase in hepatic triglyceride levels.

The *GRIN2C* gene encodes the N-methyl-D-aspartate (NMDA) receptor, a subtype of ionotropic glutamate receptor that forms a complex with the NR1 subfamily and whose main function is to act as calcium channels [50]. In the liver of the cage group, this gene was found to be upregulated. Previously, it has been reported that overexpression of this gene in liver tissue has been associated with hepatocellular hypertrophy and that NMDAR subunits can be used as biomarkers of liver cancer in murine models [51, 52]. Additionally, this gene is a glutamate receptor, a neurotransmitter whose excessive increase can induce lipid accumulation through excessive activation of NMDA receptors, which could produce metabolic disorders [53]. As for the *ACACB* gene, it has the function of converting acetyl-CoA into malonyl-CoA, a precursor for the biosynthesis of fatty acids, in addition to being bound to the outer mitochondrial membrane and maintaining the oxidation of fatty acids [54, 55]. In lipogenic tissues such as adipose tissue and liver, the *ACACB* gene is found in lower expression, since the main function of these is the storage and synthesis of fatty acids [56]. Additionally, this gene (*ACACB*) has been reported to be upregulated in adipose tissue of Fayoumi breed chickens and hens subjected to fasting, as well as in the Arbor Acres breed under heat stress conditions [57–59]. This upregulation reduces the oxidation of fatty acids, allowing the synthesis of triglycerides [55]. Together, the overexpression of the three genes *TENM2, GRIN2C,* and *ACACB* in the CC group would indicate a greater synthesis of fats in the liver, which could lead to the accumulation of these and produce hepatic steatosis [59].

Another overexpressed gene was *TMEM45BL*, a membrane protein that plays a role in cancer development and progression, as well as in liver cell differentiation [60, 61]. Furthermore, it has been reported as an upregulated gene in cases of liver cirrhosis [62]. However, at the time of this review, no reports of this gene in chickens or hens were found, so further studies are required to evaluate the effect of the *TMEM45BL* gene in the liver of laying hens. On the other hand, another of the upregulated genes in the liver of CC hens was *NOG*, an antagonist protein that binds to BMP ligands, interfering with the activation of the bone morphogenetic protein (BMP) signaling pathway [63, 64]. In chickens, this gene has been reported in granulosa cells of follicles, where the increment of its expression could be related to the proliferation or differentiation of granulosa cells and in the regeneration of the inner ear [65, 66]. Regarding the liver, the genes *ANGPTL4* and *NOG* have been reported as overexpressed in broiler chickens exposed to heat stress [16, 67]. The upregulation of the *NOG* gene in the liver could indicate an antioxidant effect, decreasing the levels of reactive oxygen species (ROS) and regulating the BMP signaling pathway that in turn regulates hepatic competence, which is the ability to respond to inducing signals [64, 68]. In the case of the *ANGPTL4* gene, it was also found to be upregulated in the CC group (log2FoldChange 1.97) and encodes for angiopoietin 4, which regulates ROS levels and energy homeostasis, and has been considered as objective to improve heat resistance in broiler chickens [67].

The *PFKFB3* gene, which encodes for the enzyme 6-phosphofructo-2-kinase/fructose-2,6-bisphosphatase 3, was found to be upregulated in the liver of hens housed in CC. The enzyme encoded by this gene has the function of activating glycolysis by regulating phosphofructokinase 1 and being a regulator of oxidative stress [69]. In hepatocyte cell lines of laying hens, overexpression of this gene has been reported in cells exposed to free fatty acids, which has been related to increased glycolysis, early activation of Ito cells, and increased expression of fibrosis markers [70]. Liver fibrosis develops after steatosis, since the activation of *PFKFB3* produces glycolytic reprogramming [70, 71]. In addition, it has been reported that steatosis in liver cells is related to oxidative stress [72]. Expression of genes related to oxidative stress was found in the liver tissue of hens housed in CC systems [73]; however, it was not determined whether the hens evaluated presented steatosis, and, at the time of sampling, the hens did not show signs of disease. Another gene in the DEGs was *CIART*, which encodes a protein called circadian rhythm-associated transcription repressor, whose translation product controls circadian rhythms and immune response, as well as the expression of genes that regulate cell cycle duration and apoptosis [74]. The gene was found upregulated in the liver of CC hens and its overexpression has been linked to alterations in circadian homeostasis or adaptation to stress, and other studies have found a negative and positive correlation with hepatic genes involved in carbohydrate, lipid, and fatty acid metabolism [75, 76].

*RHOB* (Ras homolog gene family member B) is a gene involved in multiple cellular processes such as gene transcription, apoptosis, and cell adhesion [77]. This gene has been reported to be upregulated in livestock by genotoxicity and stressors due to increased glucocorticoid levels [78]. According to the above, the upregulation of the *RHOB* gene in the liver of CC hens could indicate a cytoprotective role, since it decreases cellular apoptosis through the activation of the transcription factor NF-kB, which positively regulates the expression of antiapoptotic factors such as tumor necrosis factor receptor–associated factors (TNF) and inhibitors of apoptosis proteins (IAP) [79]. Furthermore, the upregulation of *CARD10* in CC hens reinforces the aforementioned findings by facilitating the activation of NF-kB, thereby promoting kinase activity [80]. On the other hand, the *SOCS2* gene, which encodes the cytokine signaling suppressor protein 2, was found to be upregulated in the liver of CC hens. According to Zhang, Schmidt, and Lamont [81], upregulation of *SOCS2* and *SOCS3* may play an important role in inhibiting the immune system in birds, as it attenuates the proinflammatory response and inhibits apoptosis in cardiac tissue of chickens exposed to heat stress, so it could have a similar function in the liver of hens [81, 82].

The *DNAJC12* gene, which encodes the DnaJ heat shock protein family member C12 (HSP40), was found to be upregulated, as was the *HSPA2* gene, in the CC group versus the CF system. This *DNAJC12* gene encodes a cochaperone that regulates the activity of the HSPA2 (HSP70) protein, whose function is protein quality control, *de novo* folding of amino acid chains or refolding misfolded proteins [83, 84]. The expression of this gene has been reported in heart, muscle, and spleen tissue of chickens raised in highlands and lowlands exposed to heat stress, showing an upregulation in lowland chickens, suggesting that the cells of these tissues would be presenting a response in protein metabolism to stress [85]. Furthermore, it has been shown that *DNAJC12* is upregulated in endoplasmic reticulum (ER) stress, where the binding of cochaperone to HSPA2 is enhanced, which could indicate that the ER of liver tissue from CC hens is exhibiting a stress response [86, 87]. An additional gene, *GADD45G*, was found to be upregulated in hens from the CC group and enriched in the KEGG cell cycle pathway. This gene was also detected in the liver transcriptomes response to hyperthermic stress across three distinct chicken lines: the broiler, the heat-resistant Fayoumi, and the F19 generation of a intercross genetic line [67]. The upregulation of *GADD45G* in the CC group, acting as a stress sensor, mediates cell cycle arrest and apoptosis in response to DNA damage, while actively contributing to the DNA repair process [88, 89].

Regarding to the genes that showed downregulation, the following were identified: *CTAGE1*, *BDKRB1*, *SH3RF2*, *SLC6A12*, and *IL7R*. The *CTAGE1* gene encodes an antigen that induces T-cell responses in cancer patients, and it has also been reported to have functionality in Golgi vesicle-mediated transport [90, 91]. This gene has been found expressed in thermogelled egg yolk, associated with the characteristic of resilience [92]. Furthermore, heat stress in broiler chickens alters the functionality of the ER, and its downregulation could negatively influence its functionality in vesicle transport of the ER and the Golgi apparatus [93]. It should be noted that there are no reports of the expression of this gene in liver samples, so additional studies are required to determine the function of this gene in the liver of laying hens.

Another downregulated gene was *BDKRB1*, which encodes the *BDKRB1*, whose expression is induced in inflammation and tissue injury, in addition to having a function in the calcium signaling pathway [46, 94]. Unlike the results obtained in this study, this gene has been reported to be upregulated in response to heat stress due to tissue damage, in addition to increasing cytosolic calcium levels in liver tissue [46, 95]. According to Kim et al. [46], it is considered a key gene in response to stress in broiler chickens. In ER stress, the activity of the *BDKRB1* gene has been reported, which helps to survive stressful conditions [46]. However, the negative regulation of this gene together with the *CTAGE1* gene and the positive regulation of *DNAJC12* would allow us to speculate that ER stress is occurring, and the stress response is being affected, producing a deregulation of calcium homeostasis [96, 97]. On the other hand, the *SH3RF2* gene encodes an E3 ubiquitin ligase that participates in the regulation of the intensity of apoptotic processes by modifying JNK signaling and plays a role in cell differentiation [98]. In broiler chickens and laying hens, this gene has caused productive interest, since mutations associated with high growth and body weight have been found through the regulation of appetite in the hypothalamus [99]. In this study, the expression of the *SH3RF2* gene was found to be suppressed in the liver of CC hens. According to the review carried out by the authors, the expression of this gene has not been reported in the liver of laying hens, but in mouse and monkey livers, its negative regulation allows the upregulation of genes such as *ACLY*, leading to the excessive production of triglycerides and cholesterol, which could be occurring in the liver tissue studied, in agreement with the genes *TENM2* and *GRIN2C* previously discussed [100].

The *SLC6A12* gene encodes the betaine/GABA transporter 1 (BGT1), and its function lies in the absorption of GABA and betaine through a Na+ and Cl−-dependent mechanism [101]. The expression of this gene has been evaluated in the liver of laying hens, where its mRNA is associated with the transport of amino acids such as taurine [33]. Furthermore, the expression of this gene is relevant for production, as it mediates the absorption of betaine, a supplement that improves egg production and laying rate in laying hens [101, 102]. Similar to the results of Wu et al. [33] where they reported the gene downregulated at 12 weeks of production, this gene was found downregulated in the liver of hens housed in CCs compared to those housed in CF systems at 80 weeks of age, which could indicate a decrease in amino acid transport and therefore affect egg production. Another downregulated gene was *IL7R*, which encodes for the *IL7R*, previously reported by Kim et al. [103], whose negative regulation modulates the adaptive immune response of broiler chickens subjected to heat stress. This modulation negatively affects the immune response, since *IL7R* is involved in lymphocyte development,homeostasis and, when soluble *IL7R* is generated, it regulates the extracellular bioavailability of interleukin 7 (IL-7) [104, 105]. Finally, the genes *ANGPTL4, DNAJC12, ACACB, BDKRB1, MCM2, MCM3,* and *SLC6A12* were used to validate the transcriptome results. Amplification of these genes by qPCR demonstrated that the transcriptome technique was reproducible and reliable in estimating DEG in liver samples from hens housed in two production systems [106]. This has also been demonstrated in other studies conducted in broiler chickens and laying hens [42, 103, 107, 108].

Other results found in this study included the statistically significant expression of 18 long noncoding RNAs (lncRNAs), which were found to be both upregulated and downregulated. lncRNAs are defined as non-protein-coding transcripts; however, they have been reported to be involved in protein translation and localization, RNA stability, and chromatin transcription and play roles in physiological processes such as cholesterol biosynthesis, homeostasis, cellular signal transduction, and transport pathways [109–111]. In chicken liver, the coexpression of these lncRNAs with genes located at less than 1-kb distance has been evaluated, demonstrating that they act as bidirectional promoters, specifically in cholesterol synthesis with the coexpression of the *DRCH24* gene and its lncRNA [112]. Furthermore, in laying hens exposed to heat stress, it was shown that lncRNAs are associated with heat stress, specifically with genes encoding heat shock proteins (*HSPA8, HSPH1, HSPA2,* and *DNAJA4*) [113]. On the other hand, the study of the coexpression of these lncRNAs with genes associated with fatty liver disease allows the selection of genotypes of laying hens with a lower probability of developing the disease [114]. This study did not include a coexpression analysis of lncRNAs with their neighboring genes, presenting an opportunity for future research to evaluate their functionality in the liver of hens raised in CC production systems compared to CF systems.

DEGs are data obtained from the differential expression analysis of transcriptome data, which are subsequently analyzed to obtain an overview of the changes observed when comparing the groups of interest, in this case the CC and CF groups [115]. In this study, 138 DEGs annotated in biological and molecular processes of DNA replication and repair, and circadian rhythms were obtained. Concordant results were found in the KEGG annotation, where the pathways enriched with statistical significance were DNA replication and the cell cycle. DNA replication and repair processes are fundamental for cell division, ensuring that daughter cells possess the same genetic information as the parental cell [116]. When DNA damage occurs, response pathways are activated that allow DNA repair to eliminate the damage, such as homologous recombination [117]. To maintain the genetic stability of cells, repair processes must be carried out so that cell replication continues, which can result in the fixation of a mutation or, if it persists, activate cell apoptosis [118]. The cell cycle includes four phases: G1, S, G2, corresponding to phases of cell growth, protein production, and DNA synthesis, and the M phase, which includes mitosis [119]. During the cell cycle, there are several checkpoints that allow DNA damage caused by harmful agents such as ROS to be repaired or eliminated, delaying the exit from the G2/M, G1/S, and SAC (spindle assembly) checkpoints [120].

Among the downregulated genes enriched in the KEGG cell cycle pathway, *BUB1B*, a Ser/Thr protein kinase responsible for regulating chromosome segregation, was found to be downregulated in the CC group [121]. This suggests induced mitotic spindle damage, leading to apoptosis, as cells undergoing mitosis are unable to correctly segregate chromosomes into daughter cells [122]. Furthermore, Retinoblastoma-like 1 (*RBL1*), a key regulator of the G1 to S phase transition, was downregulated in the CC group, which could lead to cell cycle arrest due to DNA damage [123, 124]. Cell division cycle 45 (*CDC45*), a gene encoding a helicase cofactor essential for DNA replication, was downregulated, with its depletion linked to delayed progression through the S-G2 phase, a pattern also observed in Ross broilers under heat stress [81].

In this study, the genes significantly expressed in the liver of the CC hen group were related to DNA replication and repair and its expression was suppressed. According to Jongen et al. [125], the suppression of genes related to DNA repair and replication promotes the accumulation of mutations and cell death, contributing to reduce the cellular capacity of DNA repair. Based on this, CC hens are affected in their cell cycle in liver tissue by oxidative stress, which can lead to cellular senescence [126]. In a study conducted in broiler chickens, it was reported that heat stress causes cell cycle disruption in pituitary gland tissue, as the oxidative stress generated can cause DNA damage [127]. On the other hand, the only enriched GO-CLC term was the MCM complex, essential for the initiation of replication in eukaryotic cells, where it acts as a DNA helicase and is made up of minichromosome maintenance proteins (MCM2-7) [128]. The *MCM* genes were downregulated in the CC group. Previously, in the liver of broiler chickens exposed to heat stress, it was reported that the genes *MCM2, MCM3, MCM4, MCM5,* and *MCM6*, associated with the term cell cycle (KEGG) and MCM complex (GO-CC), were found downregulated, since stress causes a physiological imbalance that stops the cell cycle, limiting cell growth, and replication at the G1/S checkpoint [108]. This occurs when DNA is damaged and replication is restricted to perform the necessary repairs [129]. According to the results obtained in this study, this could indicate a state of cellular instability associated with cell cycle arrest in the liver tissue of hens housed in cages [108]. This could affect egg production, since the cellular senescence produced leads to lipid metabolism disorders and metabolic diseases [130].

## 5. Conclusion

Through the analysis of the liver transcriptome of hens housed in the CC versus CF production system, genes such as *TENM2, GRIN2C, ACACB,* and *SH3RF2* were identified, which can modulate fat synthesis in the liver, indicating that the production system would produce changes in triglyceride production in birds. In addition, the CC production system would influence DNA replication and the cell cycle, since the genes related to these terms were found suppressed, indicating cellular instability that would lead to cellular senescence or apoptosis. Therefore, this study contributes to a better understanding of the metabolic pathways affected in hens housed in CCs a restrictive egg production system and the differential expression of genes between hens from the two systems that can affect the metabolism pathways of laying hens.

## Figures and Tables

**Table 1:** Sequences of primers used for the qPCR validation.

Gene		Primer sequence	Amplicon (bp)	References
*ANGPTL4*	F	CACAAGCTCCCAGAGGACTG	189	This study
	R	GTAGGCATCCCAAAGCTGGT		
*DNAJC12*	F	TGTCTGATGGGAACAGGGAGCAAG	118	
	R	CAGCGGAAACGTAAGTGCCAGTAG		
*ACACB*	F	ACTCCCATCTGCTTCAACAGCCC	132	
	R	GAAATTCAGTTCCTGCACCGTCCC		
*BDKRB1*	F	CTACAGCCAAAGGGATCTGCTTGC	160	
	R	ACCAGACGATTAGCCATTCCCCAC		
*MCM2*	F	TTGCGATCACTCAGGCAACT	158	
	R	TCCTGGTTCTGGGACTGGAA		
*MCM3*	F	ATCAGCGACAACCAGTACCGCC	194	
	R	CCCTCCAGCCCAATGTAGAAGTCC		

*SLC6A12*	F	TGCCAACCGCTTCTACGACAAC	114	Wu et al. [33]
	R	AACAAGAAGACAGCCAAGCAGAGAC		

*ACTB*	F	GCCCCCAAAGTTCTACAAT	110	Rodríguez‐Hernández et al. [22]
	R	AGGCGAGTAACTTCCTGTA		

**Table 2:** Summary of the quality of the reads.

Sample	RIN	Concentration (μg)	Raw reads	GC%	Error rate	Q20%	Q30%	Total reads filtered
CC1	6.4	68.69	49,480,118	48.19	0.03	97.91	93.8	49,256,784
CC2	6.8	108.06	50,551,146	48.22	0.03	97.59	93	50,292,888
CC3	7.6	144.82	50,819,676	48.28	0.03	97.69	93.3	50,544,382
CF1	7.2	86.64	49,861,874	47.84	0.03	97.86	93.6	49,646,028
CF2	7.6	135.17	51,480,200	47.96	0.03	98.02	94.11	51,271,664
CF3	7.2	92.11	50,190,130	47.43	0.03	97.94	93.9	49,967,170

**Table 3:** Read count data and mapping to the reference genome.

Sample	Mapping %	Uniquely mapped		Multiple mapped		Unmapped reads	
		Number	%	Number	%	Number	%
CC1	96.59	43,861,214	89.05	2,774,150	5.63	2,500,044	5.08
CC2	95.90	44,354,200	88.19	2,829,776	5.63	2,991,606	5.95
CC3	95.75	44,560,120	88.16	2,791,726	5.52	3,055,920	6.05
CF1	96.18	44,087,174	88.80	2,575,338	5.19	2,867,988	5.78
CF2	96.07	45,087,814	87.94	2,958,310	5.77	3,041,146	5.93
CF3	96.40	44,478,436	89.02	2,682,106	5.37	2,660,526	5.32

## Data Availability

The data that support the findings of this study are available in the supporting information of this article.

## Supporting Information

Supporting Information 1 Supporting Table S1: DESeq2 results table.

Supporting Information 2 Supporting Table S2: Gene Ontology (GO) enrichment results' table.

Supporting Information 3 Supporting Table S3: Kyoto Encyclopedia of Genes and Genomes (KEGG) pathway enrichment results' table.

## References

[1] Miranda J. M., Anton X., Redondo-Valbuena C. (2015). Egg and Egg-Derived Foods: Effects on Human Health and Use as Functional Foods. *Nutrients*.

[2] Ahammed M., Chae B. J., Lohakare J. (2014). Comparison of Aviary, Barn and Conventional Cage Raising of Chickens on Laying Performance and Egg Quality. *Asian-Australasian Journal of Animal Sciences*.

[3] Tainika B., Şekeroğlu A. (2020). Effect of Production Systems for Laying Hens on Hen Welfare. *Turkish Journal of Agriculture-Food Science and Technology*.

[4] Molnár S., Szőllősi L. (2020). Sustainability and Quality Aspects of Different Table Egg Production Systems: A Literature Review. *Sustainability*.

[5] Scrinis G., Parker C., Carey R. (2017). The Caged Chicken or the Free-Range Egg? the Regulatory and Market Dynamics of Layer-Hen Welfare in the UK, Australia and the USA. *Journal of Agricultural and Environmental Ethics*.

[6] De Vylder J., Dewulf J., Van Hoorebeke S. (2011). Horizontal Transmission of Salmonella Enteritidis in Groups of Experimentally Infected Laying Hens Housed in Different Housing Systems. *Poultry Science*.

[7] Lay D. C., Fulton R. M., Hester P. Y. (2011). Hen Welfare in Different Housing Systems. *Poultry Science*.

[8] von Eugen K., Nordquist R. E., Zeinstra E., van der Staay F. J. (2019). Stocking Density Affects Stress and Anxious Behavior in the Laying Hen Chick During Rearing. *Animals*.

[9] Smith S. M., Vale W. W. (2006). The Role of the Hypothalamic-Pituitary-Adrenal Axis in Neuroendocrine Responses to Stress. *Dialogues in Clinical Neuroscience*.

[10] Dunlavey C. J. (2018). Introduction to the Hypothalamic-Pituitary-Adrenal Axis: Healthy and Dysregulated Stress Responses, Developmental Stress and Neurodegeneration. *Journal of Undergraduate Neuroscience Education: JUNE: A Publication of FUN, Faculty for Undergraduate Neuroscience*.

[11] Lara L. J., Rostagno M. H. (2013). Impact of Heat Stress on Poultry Production. *Animals*.

[12] Simitzis P. E., Kalogeraki E., Goliomytis M. (2012). Impact of Stocking Density on Broiler Growth Performance, Meat Characteristics, Behavioural Components and Indicators of Physiological and Oxidative Stress. *British Poultry Science*.

[13] Selvam R., Saravanakumar M., Suresh S., Sureshbabu G., Sasikumar M., Prashanth D. (2017). Effect of Vitamin E Supplementation and High Stocking Density on the Performance and Stress Parameters of Broilers. *Revista Brasileira de Ciência Avícola*.

[14] Najafi P., Zulkifli I., Amat Jajuli N. (2015). Environmental Temperature and Stocking Density Effects on Acute Phase Proteins, Heat Shock Protein 70, Circulating Corticosterone and Performance in Broiler Chickens. *International Journal of Biometeorology*.

[15] Nasr M. A. F., Alkhedaide A. Q., Ramadan A. A. I., Hafez A. E. S. E., Hussein M. A. (2021). Potential Impact of Stocking Density on Growth, Carcass Traits, Indicators of Biochemical and Oxidative Stress and Meat Quality of Different Broiler Breeds. *Poultry Science*.

[16] Emami N. K., Jung U., Voy B., Dridi S. (2020). Radical Response: Effects of Heat Stress-Induced Oxidative Stress on Lipid Metabolism in the Avian Liver. *Antioxidants*.

[17] Weimer S. L., Wideman R. F., Scanes C. G., Mauromoustakos A., Christensen K. D., Vizzier-Thaxton Y. (2018). An Evaluation of Methods for Measuring Stress in Broiler Chickens. *Poultry Science*.

[18] Nwaigwe C. U., Ihedioha J. I., Shoyinka S. V., Nwaigwe C. O. (2020). Evaluation of the Hematological and Clinical Biochemical Markers of Stress in Broiler Chickens. *Veterinary World*.

[19] Weimer S. L., Wideman R. F., Scanes C. G., Mauromoustakos A., Christensen K. D., Vizzier-Thaxton Y. (2020). Broiler Stress Responses to Light Intensity, Flooring Type, and Leg Weakness as Assessed by Heterophil-To-Lymphocyte Ratios, Serum Corticosterone, Infrared Thermography, and Latency to Lie. *Poultry Science*.

[20] Perini F., Cendron F., Rovelli G., Castellini C., Cassandro M., Lasagna E. (2020). Emerging Genetic Tools to Investigate Molecular Pathways Related to Heat Stress in Chickens: A Review. *Animals*.

[21] Casamassimi A., Federico A., Rienzo M., Esposito S., Ciccodicola A. (2017). Transcriptome Profiling in Human Diseases: New Advances and Perspectives. *International Journal of Molecular Sciences*.

[22] Rodríguez-Hernández R., Oviedo-Rondón E. O., Rondón-Barragán I. S. (2021). Identification of Reliable Reference Genes for Expression Studies in the Magnum of Laying Hens Housed in Cage and Cage-free Systems. *Veterinary medicine and science*.

[23] Andrews S. FastQC A Quality Control Tool for High Throughput Sequence Data. https://www.bioinformatics.babraham.ac.uk/projects/fastqc/.

[24] Krueger F. (2021). Trim Galore. *GitHub Repository*.

[25] Kim D., Langmead B., Salzberg S. L. (2015). HISAT: A Fast Spliced Aligner With Low Memory Requirements. *Nature Methods*.

[26] Liao Y., Smyth G. K., Shi W. (2014). featureCounts: An Efficient General Purpose Program for Assigning Sequence Reads to Genomic Features. *Bioinformatics*.

[27] Risso D., Ngai J., Speed T. P., Dudoit S. (2014). Normalization of RNA-Seq Data Using Factor Analysis of Control Genes or Samples. *Nature Biotechnology*.

[28] Love M. I., Huber W., Anders S. (2014). Moderated Estimation of Fold Change and Dispersion for RNA-Seq Data With DESeq2. *Genome Biology*.

[29] Benjamini Y., Drai D., Elmer G., Kafkafi N., Golani I. (2001). Controlling the False Discovery Rate in Behavior Genetics Research. *Behavioural Brain Research*.

[30] Yu G., Wang L. G., Han Y., He Q. Y. (2012). clusterProfiler: An R Package for Comparing Biological Themes Among Gene Clusters. *OMICS: A Journal of Integrative Biology*.

[31] Livak K. J., Schmittgen T. D. (2001). Analysis of Relative Gene Expression Data Using Real-Time Quantitative PCR and the 2−ΔΔCT Method. *Methods*.

[32] Herrera-Sánchez M. P., Lozano-Villegas K. J., Rondón-Barragán I. S., Rodríguez-Hernández R. (2023). Identification of Reference Genes for Expression Studies in the Liver and Spleen of Laying Hens Housed in Cage and Cage-Free Systems. *Open Veterinary Journal*.

[33] Wu J., Wang Y., An Y. (2024). Identification of Genes Related to Growth and Amino Acid Metabolism From the Transcriptome Profile of the Liver of Growing Laying Hens. *Poultry Science*.

[34] Khumput S., Thiengtham J. (2020). Behavior, Egg Production, and Bone Strength of Commercial Laying Hens at Various Cage Densities and Different Cage Types. *Songklanakarin Journal of Science and Technology*.

[35] Wan Y., Guan H., Wang D. (2023). Effects of Cage Stocking Density on the Production Performance, Serum Biochemistry, Immune Level, and Intestinal Morphology of 2 Laying Hen Breeds. *The Journal of Applied Poultry Research*.

[36] Xu W., Song Z., Wang W. (2022). Effects of in Ovo Feeding of T10, C12-Conjugated Linoleic Acid on Hepatic Lipid Metabolism and Subcutaneous Adipose Tissue Deposition in Newly Hatched Broiler Chicks. *Poultry Science*.

[37] Zhu M. K., Li H. Y., Bai L. H., Wang L. S., Zou X. T. (2020). Histological Changes, Lipid Metabolism, and Oxidative and Endoplasmic Reticulum Stress in the Liver of Laying Hens Exposed to Cadmium Concentrations. *Poultry Science*.

[38] Magnuson A. D., Liu G., Sun T. (2020). Supplemental Methionine and Stocking Density Affect Antioxidant Status, Fatty Acid Profiles, and Growth Performance of Broiler Chickens. *Journal of Animal Science*.

[39] Ma B., Xing T., Li J., Zhang L., Jiang Y., Gao F. (2022). Chronic Heat Stress Causes Liver Damage via Endoplasmic Reticulum Stress-Induced Apoptosis in Broilers. *Poultry Science*.

[40] Barreto Sánchez A. L., Wang Q., Thiam M. (2022). Liver Transcriptome Response to Heat Stress in Beijing You Chickens and Guang Ming Broilers. *Genes*.

[41] Kumar H., Iskender A. U., Srikanth K. (2019). Transcriptome of Chicken Liver Tissues Reveals the Candidate Genes and Pathways Responsible for Adaptation into Two Different Climatic Conditions. *Animals*.

[42] Kim D. Y., Lim B., Kim J. M., Kil D. Y. (2022). Integrated Transcriptome Analysis for the Hepatic and Jejunal Mucosa Tissues of Broiler Chickens Raised Under Heat Stress Conditions. *Journal of Animal Science and Biotechnology*.

[43] Sprang M., Andrade-Navarro M. A., Fontaine J. F. (2022). Batch Effect Detection and Correction in RNA-Seq Data Using Machine-Learning-Based Automated Assessment of Quality. *BMC Bioinformatics*.

[44] Höglund A., Strempfl K., Fogelholm J., Wright D., Henriksen R. (2020). The Genetic Regulation of Size Variation in the Transcriptome of the Cerebrum in the Chicken and its Role in Domestication and Brain Size Evolution. *BMC Genomics*.

[45] Wolthuis J. C., Magnúsdóttir S., Stigter E. (2023). Multi-Country Metabolic Signature Discovery for Chicken Health Classification. *Metabolomics*.

[46] Kim H., Kim H., Seong P. (2021). Transcriptomic Response under Heat Stress in Chickens Revealed the Regulation of Genes and Alteration of Metabolism to Maintain Homeostasis. *Animals*.

[47] Schroyen M., Lesuisse J., Lamberigts C. (2021). Direct and Maternal Reduced Balanced Protein Diet Influences the Liver Transcriptome in Chickens. *British Journal of Nutrition*.

[48] Ayling R., Marshall W., Lapsley M., Day A. P., Ayling R. (2014). Clinical Biochemistry of Nutrition. *Clinical Biochemistry: Metabolic and Clinical Aspects*.

[49] O’Farrell F., Aleyakpo B., Mustafa R. (2023). Evidence for Involvement of the Alcohol Consumption WDPCP Gene in Lipid Metabolism, and Liver Cirrhosis. *Scientific Reports*.

[50] Nemoto K., Tanaka T., Ikeda A. (2011). Super-Induced Gene Expression of the N-Methyl-D-Aspartate Receptor 2C Subunit in Chemical-Induced Hypertrophic Liver in Rats. *Journal of Toxicological Sciences*.

[51] Nemoto K., Ikeda A., Tanaka T. (2013). Change in the Gene Expression of the *N*-Methyl-D-Aspartate Receptor 2C Subunit by Dietary β-naphthoflavone, Indole-3-Carbinol, or Acetaminophen in the Rat Liver. *Journal of Toxicological Sciences*.

[52] Stepanov Y. V., Golovynska I., Dziubenko N. V. (2022). NMDA Receptor Expression During Cell Transformation Process at Early Stages of Liver Cancer in Rodent Models. *American Journal of Physiology-Gastrointestinal and Liver Physiology*.

[53] Huang X. T., Yang J. X., Wang Z. (2021). Activation of N-Methyl-D-Aspartate Receptor Regulates Insulin Sensitivity and Lipid Metabolism. *Theranostics*.

[54] Im S. S., Hammond L. E., Yousef L. (2009). Sterol Regulatory Element Binding Protein 1a Regulates Hepatic Fatty Acid Partitioning by Activating Acetyl Coenzyme A Carboxylase 2. *Molecular and Cellular Biology*.

[55] Prasad C. S., Vinoo R., Chatterjee R. N. (2021). Expression Profile of Acetyl CoA Carboxylase Beta (ACACB) Gene During the Pre and Post-Hatch Period in Chicken. *Indian Journal of Animal Research*.

[56] Abu-Elheiga L., Matzuk M. M., Abo-Hashema K. A., Wakil S. J. (2001). Continuous Fatty Acid Oxidation and Reduced Fat Storage in Mice Lacking Acetyl-CoA Carboxylase 2. *Science (New York, N.Y.)*.

[57] Ji B., Ernest B., Gooding J. R. (2012). Transcriptomic and Metabolomic Profiling of Chicken Adipose Tissue in Response to Insulin Neutralization and Fasting. *BMC Genomics*.

[58] Ji B., Middleton J. L., Ernest B. (2014). Molecular and Metabolic Profiles Suggest That Increased Lipid Catabolism in Adipose Tissue Contributes to Leanness in Domestic Chickens. *Physiological Genomics*.

[59] Lu Z., He X. F., Ma B. B. (2019). Increased Fat Synthesis and Limited Apolipoprotein B Cause Lipid Accumulation in the Liver of Broiler Chickens Exposed to Chronic Heat Stress. *Poultry Science*.

[60] Ghosheh N., Küppers-Munther B., Asplund A. (2017). Comparative Transcriptomics of Hepatic Differentiation of Human Pluripotent Stem Cells and Adult Human Liver Tissue. *Physiological Genomics*.

[61] Marx S., Dal Maso T., Chen J. W. (2020). Transmembrane (TMEM) Protein Family Members: Poorly Characterized Even if Essential for the Metastatic Process. *Seminars in Cancer Biology*.

[62] Chan K. M., Wu T. H., Wu T. J., Chou H. S., Yu M. C., Lee W. C. (2016). Bioinformatics Microarray Analysis and Identification of Gene Expression Profiles Associated With Cirrhotic Liver. *The Kaohsiung Journal of Medical Sciences*.

[63] Krause C., Guzman A., Knaus P. (2011). Noggin. *The International Journal of Biochemistry & Cell Biology*.

[64] Polyzos S. A., Kountouras J., Anastasilakis A. D. (2018). Noggin Levels in Nonalcoholic Fatty Liver Disease: The Effect of Vitamin E Treatment. *Hormones*.

[65] Jiang L., Xu J., Jin R. (2018). Transcriptomic Analysis of Chicken Cochleae After Gentamicin Damage and the Involvement of Four Signaling Pathways (Notch, FGF, Wnt and BMP) in Hair Cell Regeneration. *Hearing Research*.

[66] Gong Y., Li D., Sun Y., Kang L., Jiang Y. (2023). Expression and Regulation of Noggin4 Gene in Chicken Ovarian Follicles and Its Role in the Proliferation and Differentiation of Granulosa Cells. *Theriogenology*.

[67] Lan X., Hsieh J. C., Schmidt C. J., Zhu Q., Lamont S. J. (2016). Liver Transcriptome Response to Hyperthermic Stress in Three Distinct Chicken Lines. *BMC Genomics*.

[68] Shin D., Monga S. P. (2013). Cellular and Molecular Basis of Liver Development. *Comprehensive Physiology*.

[69] Lake J. A., Abasht B. (2020). Glucolipotoxicity: A Proposed Etiology for Wooden Breast and Related Myopathies in Commercial Broiler Chickens. *Frontiers in Physiology*.

[70] Huang C., Gao X., Shi Y. (2022). Inhibition of Hepatic AMPK Pathway Contributes to Free Fatty Acids-Induced Fatty Liver Disease in Laying Hen. *Metabolites*.

[71] Mejias M., Gallego J., Naranjo-Suarez S. (2020). CPEB4 Increases Expression of PFKFB3 to Induce Glycolysis and Activate Mouse and Human Hepatic Stellate Cells, Promoting Liver Fibrosis. *Gastroenterology*.

[72] Vasileva L. V., Savova M. S., Amirova K. M., Dinkova-Kostova A. T., Georgiev M. I. (2020). Obesity and NRF2-Mediated Cytoprotection: Where Is the Missing Link?. *Pharmacological Research*.

[73] Herrera-Sánchez M. P., Rodríguez-Hernández R., Rondón-Barragán I. S. (2024). Stress-Related Gene Expression in Liver Tissues From Laying Hens Housed in Conventional Cage and Cage-Free Systems in the Tropics. *Veterinary Medicine International*.

[74] Alenezi H., Ozkan J., Willcox M., Parnell G., Carnt N. (2022). Differential Gene Expression of the Healthy Conjunctiva During the Day. *Contact Lens and Anterior Eye*.

[75] Reshetnikov V. V., Kisaretova P. E., Ershov N. I., Merkulova T. I., Bondar N. P. (2021). Social Defeat Stress in Adult Mice Causes Alterations in Gene Expression, Alternative Splicing, and the Epigenetic Landscape of H3K4me3 in the Prefrontal Cortex: An Impact of Early-Life Stress. *Progress in Neuro-Psychopharmacology and Biological Psychiatry*.

[76] Yan R., Wang K., Wang Q. (2022). Probiotic Lactobacillus Casei Shirota Prevents Acute Liver Injury by Reshaping the Gut Microbiota to Alleviate Excessive Inflammation and Metabolic Disorders. *Microbial Biotechnology*.

[77] Giannuzzi D., Biolatti B., Longato E. (2021). Application of RNA-Sequencing to Identify Biomarkers in Broiler Chickens Prophylactic Administered With Antimicrobial Agents. *Animal*.

[78] Sajjanar B., Aalam M. T., Khan O. (2023). Genome-Wide Expression Analysis Reveals Different Heat Shock Responses in Indigenous (*Bos indicus*) and Crossbred (*Bos indicus* X *Bos taurus*) Cattle. *Genes and Environment*.

[79] Li Y. D., Liu Y. P., Cao D. M. (2011). Induction of Small G Protein RhoB by Non-Genotoxic Stress Inhibits Apoptosis and Activates NF-Κb. *Journal of Cellular Physiology*.

[80] Liu X., Lu R., Xia Y., Wu S., Sun J. (2010). Eukaryotic Signaling Pathways Targeted by Salmonella Effector Protein AvrA in Intestinal Infection In Vivo. *BMC Microbiology*.

[81] Zhang J., Schmidt C. J., Lamont S. J. (2017). Transcriptome Analysis Reveals Potential Mechanisms Underlying Differential Heart Development in Fast- and Slow-Growing Broilers Under Heat Stress. *BMC Genomics*.

[82] Jo D., Liu D., Yao S., Collins R. D., Hawiger J. (2005). Intracellular Protein Therapy With SOCS3 Inhibits Inflammation and Apoptosis. *Nature Medicine*.

[83] Gallego D., Leal F., Gámez A. (2020). Pathogenic Variants of DNAJC12 and Evaluation of the Encoded Cochaperone as a Genetic Modifier of Hyperphenylalaninemia. *Human Mutation*.

[84] Zhang H., Gong W., Wu S., Perrett S. (2022). Hsp70 in Redox Homeostasis. *Cells*.

[85] Te Pas M. F. W., Park W., Srikanth K. (2019). Transcriptomic Profiles of Muscle, Heart, and Spleen in Reaction to Circadian Heat Stress in Ethiopian Highland and Lowland Male Chicken. *Cell Stress & Chaperones*.

[86] Choi J., Djebbar S., Fournier A., Labrie C. (2014). The Co-Chaperone DNAJC12 Binds to Hsc70 and Is Upregulated by Endoplasmic Reticulum Stress. *Cell Stress & Chaperones*.

[87] Li Y., Li M., Jin F., Liu J., Chen M., Yin J. (2021). DNAJC12 Promotes Lung Cancer Growth by Regulating the Activation of β-Catenin. *International Journal of Molecular Medicine*.

[88] Zeng L. P., Qin Y. Q., Lu X. M., Feng Z. B., Fang X. L. (2023). Identify GADD45G as a Potential Target of 4-Methoxydalbergione in Treatment of Liver Cancer: Bioinformatics Analysis and In Vivo Experiment. *World Journal of Surgical Oncology*.

[89] Salvador J. M., Brown-Clay J. D., Fornace A. J., Liebermann D., Hoffman B. (2013). Gadd45 in Stress Signaling, Cell Cycle Control, and Apoptosis. *Advances in Experimental Medicine and Biology*.

[90] Litvinov I. V., Cordeiro B., Huang Y. (2014). Ectopic Expression of Cancer-Testis Antigens in Cutaneous T-Cell Lymphoma Patients. *Clinical Cancer Research*.

[91] Schließer P., Struebing F. L., Northoff B. H. (2023). Detection of a Parkinson’s Disease-Specific MicroRNA Signature in Nasal and Oral Swabs. *Movement Disorders*.

[92] Zhang R., Li X., Ma Y. (2023). Identification of Candidate Genomic Regions for Thermogelled Egg Yolk Traits Based on a Genome-Wide Association Study. *Poultry Science*.

[93] Tokutake Y., Takanashi R., Kikusato M., Toyomizu M., Sato K. (2022). Effect of Dietary 4-Phenylbuthyric Acid Supplementation on Acute Heat-Stress-Induced Hyperthermia in Broiler Chickens. *Animals*.

[94] Garner J. B., Chamberlain A. J., Vander Jagt C. (2020). Gene Expression of the Heat Stress Response in Bovine Peripheral White Blood Cells and Milk Somatic Cells In Vivo. *Scientific Reports*.

[95] Coble D. J., Fleming D., Persia M. E. (2014). RNA-Seq Analysis of Broiler Liver Transcriptome Reveals Novel Responses to High Ambient Temperature. *BMC Genomics*.

[96] Punapart M., Seppa K., Jagomäe T. (2021). The Expression of RAAS Key Receptors, *Agtr2* and *Bdkrb1*, Is Downregulated at an Early Stage in a Rat Model of Wolfram Syndrome. *Genes*.

[97] Chen X., Shi C., He M., Xiong S., Xia X. (2023). Endoplasmic Reticulum Stress: Molecular Mechanism and Therapeutic Targets. *Signal Transduction and Targeted Therapy*.

[98] Ropka-Molik K., Stefaniuk-Szmukier M., Piórkowska K., Szmatoła T., Bugno-Poniewierska M. (2018). Molecular Characterization of the Apoptosis-Related SH3RF1 and SH3RF2 Genes and Their Association With Exercise Performance in Arabian Horses. *BMC Veterinary Research*.

[99] Jing Z., Wang X., Cheng Y. (2020). Detection of CNV in the SH3RF2 Gene and Its Effects on Growth and Carcass Traits in Chickens. *BMC Genetics*.

[100] Yang X., Sun D., Xiang H. (2021). Hepatocyte SH3RF2 Deficiency Is a Key Aggravator for NAFLD. *Hepatology*.

[101] Kempson S. A., Zhou Y., Danbolt N. C. (2014). The Betaine/GABA Transporter and Betaine: Roles in Brain, Kidney, and Liver. *Frontiers in Physiology*.

[102] Abd El-Ghany W. A., Babazadeh D. (2022). Betaine: A Potential Nutritional Metabolite in the Poultry Industry. *Animals*.

[103] Kim D. Y., Han G. P., Lim C., Kim J. M., Kil D. Y. (2023). Effect of Dietary Betaine Supplementation on the Liver Transcriptome Profile in Broiler Chickens Under Heat Stress Conditions. *Animal bioscience*.

[104] Berndt A., Pieper J., Methner U. (2006). Circulating γδ T Cells in Response to*Salmonellaenterica*Serovar Enteritidis Exposure in Chickens. *Infection and Immunity*.

[105] Ampuero S., Bahamonde G., Tempio F. (2022). IL-7/IL7R axis Dysfunction in Adults With Severe Community-Acquired Pneumonia (CAP): A Cross-Sectional Study. *Scientific Reports*.

[106] Coenye T. (2021). Do Results Obtained With RNA-Sequencing Require Independent Verification?. *Biofilms*.

[107] Wang Y., Jia X., Hsieh J. C. F. (2021). Transcriptome Response of Liver and Muscle in Heat-Stressed Laying Hens. *Genes*.

[108] Lim C., Lim B., Kil D. Y., Kim J. M. (2022). Hepatic Transcriptome Profiling According to Growth Rate Reveals Acclimation in Metabolic Regulatory Mechanisms to Cyclic Heat Stress in Broiler Chickens. *Poultry Science*.

[109] Deforges J., Reis R. S., Jacquet P. (2019). Control of Cognate Sense mRNA Translation by Cis-Natural Antisense RNAs. *Plant Physiology*.

[110] Pisignano G., Ladomery M. (2021). Epigenetic Regulation of Alternative Splicing: How LncRNAs Tailor the Message. *Non-Coding RNA*.

[111] Mattick J. S., Amaral P. P., Carninci P. (2023). Long Non-Coding RNAs: Definitions, Functions, Challenges and Recommendations. *Nature Reviews Molecular Cell Biology*.

[112] Muret K., Klopp C., Wucher V. (2017). Long Noncoding RNA Repertoire in Chicken Liver and Adipose Tissue. *Genetics Selection Evolution*.

[113] Wu X., Du X., Pian H., Yu D. (2024). Effect of Curcumin on Hepatic mRNA and lncRNA Co-Expression in Heat-Stressed Laying Hens. *International Journal of Molecular Sciences*.

[114] Chamani R., Masoudi A. A., Vaez Torshizi R., Alipour S. (2024). Long Non-Coding RNAs Induce Fatty Liver During Developmental Stages in Laying Hen. *Journal of Agricultural Science and Technology A*.

[115] Hekman J. P., Johnson J. L., Kukekova A. V. (2016). Transcriptome Analysis in Domesticated Species: Challenges and Strategies. *Bioinformatics and Biology Insights*.

[116] Jarillo J., Ibarra B., Cao-García F. J. (2021). DNA Replication: *In Vitro* Single-Molecule Manipulation Data Analysis and Models. *Computational and Structural Biotechnology Journal*.

[117] Chatterjee N., Walker G. C. (2017). Mechanisms of DNA Damage, Repair, and Mutagenesis. *Environmental and Molecular Mutagenesis*.

[118] Hakem R. (2008). DNA-Damage Repair; The Good, the Bad, and the Ugly. *The EMBO Journal*.

[119] Wang Z. (2021). Regulation of Cell Cycle Progression by Growth Factor-Induced Cell Signaling. *Cells*.

[120] Xu Y., Jiao Y., Liu C. (2024). R-Loop and Diseases: The Cell Cycle Matters. *Molecular Cancer*.

[121] Hu S., Song Y., Li X. (2024). Comparative Transcriptomics Analysis Identifies Crucial Genes and Pathways During Goose Spleen Development. *Frontiers in Immunology*.

[122] Hicks J. A., Pike B. E., Liu H. C. (2022). Alterations in Hepatic Mitotic and Cell Cycle Transcriptional Networks During the Metabolic Switch in Broiler Chicks. *Frontiers in Physiology*.

[123] Ventura E., Iannuzzi C. A., Pentimalli F., Giordano A., Morrione A. (2021). RBL1/p107 Expression Levels Are Modulated by Multiple Signaling Pathways. *Cancers*.

[124] Rengaraj D., Lee B. R., Choi J. W. (2012). Gene Pathways and Cell Cycle-Related Genes in Cultured Avian Primordial Germ Cells. *Poultry Science*.

[125] Jongen J. M. J., van der Waals L. M., Trumpi K. (2017). Downregulation of DNA Repair Proteins and Increased DNA Damage in Hypoxic Colon Cancer Cells Is a Therapeutically Exploitable Vulnerability. *Oncotarget*.

[126] Collin G., Huna A., Warnier M., Flaman J. M., Bernard D. (2018). Transcriptional Repression of DNA Repair Genes Is a Hallmark and a Cause of Cellular Senescence. *Cell Death & Disease*.

[127] Pritchett E. M., Van Goor A., Schneider B. K., Young M., Lamont S. J., Schmidt C. J. (2023). Chicken Pituitary Transcriptomic Responses to Acute Heat Stress. *Molecular Biology Reports*.

[128] Dong Y., Newman M., Pederson S. M., Barthelson K., Hin N., Lardelli M. (2021). Transcriptome Analyses of 7-Day-Old Zebrafish Larvae Possessing a Familial Alzheimer’s Disease-Like Mutation in Psen1 Indicate Effects on Oxidative Phosphorylation, ECM and MCM Functions, and Iron Homeostasis. *BMC Genomics*.

[129] Sun L., Lamont S. J., Cooksey A. M. (2015). Transcriptome Response to Heat Stress in a Chicken Hepatocellular Carcinoma Cell Line. *Cell Stress & Chaperones*.

[130] Gu Y. F., Chen Y. P., Jin R., Wang C., Wen C., Zhou Y. M. (2021). Age-Related Changes in Liver Metabolism and Antioxidant Capacity of Laying Hens. *Poultry Science*.

